# Peer mentoring for individuals with an eating disorder: a qualitative evaluation of a pilot program

**DOI:** 10.1186/s40337-020-00301-8

**Published:** 2020-07-01

**Authors:** Freya Hanly, Benjamin Torrens-Witherow, Narelle Warren, David Castle, Andrea Phillipou, Jennifer Beveridge, Zoe Jenkins, Richard Newton, Leah Brennan

**Affiliations:** 1 School of Behavioural and Health Science, Australian Catholic University, Melbourne, VIC Australia; 2 School of Social Sciences, Monash University, Melbourne, VIC Australia; 3 Department of Psychiatry, St Vincent’s Hospital, Melbourne, VIC Australia; 4 Department of Psychiatry, The University of Melbourne, Melbourne, VIC Australia; 5 Centre for Mental Health, Swinburne University of Technology, PO Box 218, Hawthorn, VIC 3122 Australia; 6 Department of Mental Health, Austin Health, Melbourne, VIC Australia; 7 Eating Disorders Victoria, Melbourne, VIC Australia; 8 Department of Psychiatry, Monash University, Melbourne, Victoria Australia; 9 Peninsula Health, Frankston, Victoria Australia; 10 Centre for Eating, Weight and Body Image, Melbourne, VIC Australia; 11 School of Psychology and Public Health, La Trobe University, Wodonga, VIC Australia

**Keywords:** Eating disorder, Peer mentoring, Peer work, Anorexia nervosa, Treatment

## Abstract

**Background:**

After receiving intensive medical treatment; individuals with eating disorders often require ongoing care to maintain their recovery, build social networks, and reduce risk of relapse.

**Methods:**

To address this important transition period, a six-month peer mentoring program was developed and piloted in Melbourne, Australia. Twelve adults with a past history of an eating disorder (mentors) were paired with 14 individuals with a current eating disorder (mentees). Pairs met for thirteen mentoring sessions in community settings. Throughout the program mentees and mentors completed reflective questions online. Upon completion of the program, qualitative interviews were conducted. Both online reflections and interviews explored themes relating to perceived benefits and challenges of participation in the peer mentoring program, and the differences between mentoring and traditional treatment.

**Results:**

Thematic analysis identified several benefits for mentees; including hope, reconnection with others, and re-engaging with the world. The majority of mentees described their mentor as uniquely supportive due to their past experience of an eating disorder. Mentors reported experiencing benefits such as increased connection with self and others, and indicated that the experience helped them positively reframe their past experience of an eating disorder. Ending the relationship at the completion of the program was a significant challenge for both groups, and managing boundaries was deemed a main challenge by mentors.

**Conclusions:**

Overall, results indicated that this mode of informal support may be worthy of further investigation as an adjunct to clinical treatment programs for this population.

**Trial registration:**

Australian and New Zealand Clinical Trials registration number - ACTRN12617001412325 - Date of registration – 05/10/2017 (Retrospectively registered)

## Plain English summary

Individuals recovered from an eating disorder (‘mentors’) and individuals currently with an eating disorder (‘mentees’) participated in a peer mentoring program. Thirteen mentoring sessions occurred in community settings. Participants were asked to complete online reflections at three time-points: after session one, at the half-way point of the program, and at the completion of mentoring sessions. At the completion of the program, semi-structured interviews were conducted. The research identified many positive aspects relating to the experience of participation, as well as aspects of the program that could be refined in the future. Mentees noted that the support of their mentor allowed them to gain renewed hope in their recovery and helped them to re-engage socially, whilst mentors experienced benefits such as increased connection with self and others, and noted the experience helped them appreciate the wisdom they had gained from their past eating disorder. Ending the relationship at the completion of the program was a significant challenge for both groups, and managing boundaries was deemed a main challenge by mentors. Overall, the results are encouraging, but further research is required to extend and expand our knowledge about the role of peer support in the recovery journey of people with an eating disorder.

## Introduction

Eating disorders are typically chronic [1] and difficult to treat [2, 3]. They are associated with reduced quality of life [4], loss of careers [5], and social isolation [4, 6]; in addition to serious, long-term medical ramifications [7] including increased mortality [8]. A substantial proportion of individuals with eating disorders do not achieve a sustained recovery [3, 9]. Relapse rates across eating disorders are estimated at between 22 and 60% [10]. Within this context, it is important for researchers and clinicians to identify points in the recovery journey at which meaningful interventions can be applied to improve outcomes.

Recovered individuals have reported that social support is particularly pertinent after undergoing intensive treatments such as inpatient programs [11–13]. Developing supportive relationships and a sense of belonging have been identified as important factors in sustained recovery from eating disorders [12, 14, 15], and have been found to impact positively upon motivation for recovery [16].

One framework which emphasises building social support is the peer-mentoring model of mental health care [17]. This model consists of matching individuals who are in early recovery from a mental illness (mentees) to those who are further along the path of recovery [18] (mentors). Mentors typically provide practical assistance and moral support to mentees, while also fostering social support and connection [19]. The peer-mentoring model is founded on the mentor’s lived experience of the mental illness, enabling them to share hope, empathy, and life skills with the mentee [19–21]. Programs delivering peer-mentoring in addition to treatment-as-usual to individuals with schizophrenia and bipolar disorder have most commonly been reported. Research examining such programs suggest that benefits include reduced hospital days [22, 23], increased treatment engagement [24, 25], hope for recovery [26], and improved self-esteem for mentees [27].

Previous research has identified both benefits and risks for mentors who are providing such services. Meta-syntheses of research describing existing peer mentoring programs for mental health conditions have identified benefits including enhanced self-esteem and confidence [28], improved responsibility and purpose through a reframing of identity from healthcare consumer to healthcare provider [29], and improvements in social and occupational functioning [30]. Risks for mentors include job stress, burnout, and relapse [18, 19, 31]. Commonly reported difficulties include a lack of role clarity and formal expectations, uncertainty around the longevity of the mentoring role, and poor acceptance of the mentors by traditional mental health workers [28, 32–34].

There is growing interest in the use of peer mentoring as a potential addition to mental health treatment in the domain of eating disorders; however, to date few peer-mentoring programs have been implemented and evaluated in this clinical population [35]. The most recent systematic review and synthesis of this field by Fogarty et al. [11] yielded only four eligible studies (*N* = 270*)*, with only two of these [36, 37] specifically targeting clinical (as opposed to sub-clinical) eating disorders.

Perez, Van Diest, and Cutts [37] evaluated a mentoring program and found that mentees who were actively engaged in mentoring reported higher health-care treatment adherence compared to those who were yet to be matched to a mentor. Frequency of contact with a mentor was positively correlated with several quality of life domains, including better family dynamics and improvement in other close relationships; higher physical, psychological, and emotional well-being; and greater optimism for the future. Mentors reported improvements in recovery skills, were reminded of the steps toward recovery, and gained a better perspective on how far they had come in their own recovery journey. Over 90% of mentors reported that their recovery had been positively impacted by their provision of peer support.

In a qualitative study by Lippi [36], six mentees in early stages of recovery from an ED were matched with six mentors and met one-on-one weekly for six weeks. Mentees reported the program helped them develop a new (non-eating disorder) identity, increased their awareness that recovery is a process, and gave them the opportunity to re-learn social skills in a safe environment. They also reported the program was less rigid and more fun than traditional treatment. Finally, mentees reported feeling more socially engaged and more hopeful of recovery because of their relationship with their mentor. Mentors reported benefits including increased self-discovery, self-acceptance, reinforcement of the coping skills used in their own recovery, and an increased sense of purpose.

In a quantitative proof-of-concept pilot study, Ramjan, Hay, and Fogarty [38] reported on a program which matched 10 female mentees with a current ED diagnosis with 10 recovered female mentors. Pairs took part in a 13-week mentoring program. Hope, as measured by the Domain Specific Hope Scale, increased for mentees from baseline to post-program. However, all other measures used (e.g. the Eating Disorder Quality of Life scale) showed no change. The program was found to be safe for mentors, with no significant decreases in measures of general or ED-specific quality of life. A similar feasibility study [39] matched five females who had recovered from Anorexia Nervosa (AN; mentors) with six females who were recovering and in transition from inpatient care for AN (mentees). Qualitative analysis provided preliminary evidence that peer support met a clear need for people who were in the process of recovering from AN. Key themes that emerged included the sense of connection mentees felt with their mentor, the motivation and challenge mentors provided to mentees, and increased hope (mentees), which was attributed to the strength and resilience modelled by mentors. Mentors experienced benefits of increased self-awareness, and turning a negative past experience into something positive for others.

There are several key concepts of peer-mentoring in eating disorders which have not been explored in detail by the existing literature. First, it is important to investigate perceived barriers and challenges to participating in a peer-mentoring program for both mentees and mentors so that future programs can anticipate these and adapt accordingly. Second, risks in delivering peer mentoring for mentors with past EDs have yet to be explored in detail. Third, little has been reported regarding perceived differences between peer-mentoring and clinical treatment, and how the mutual delivery of these two modes of care is experienced. Moreover, it is important to ascertain from the clients’ perspective whether peer-mentoring is offering a unique aspect of care that can work alongside, and add value to, traditional treatment models.

To address the shortcomings of the research noted above, a study of the feasibility and acceptability of a six-month, one-to-one peer-mentoring program (PMP) for people with eating disorders was conducted (for detailed results, see [40]). It was found that mentees involved in the program demonstrated improvements in ED symptomatology, quality of life, mood, and perceived disability. Mentors demonstrated significant increases in certain domains of eating disorder symptomatology (i.e., in the areas of eating and weight concern); but were not in the pathological range for ED symptoms at any time point, and demonstrated no worsening of quality of life, mood, or perceived disability. Qualitative data from the study was collected to provide further context for the above findings and is the focus of the current research.

Using qualitative methodology, the current study aimed to explore both mentees’ and mentors’ perceived benefits and challenges associated with participation in a six-month, one-to-one peer-mentoring program. The research also aimed to explore perceptions among mentees regarding the differences between peer-mentoring and clinical treatment, in order to understand any unique features peer mentoring might add to recovery support.

## Method

The current study constitutes the qualitative component of a larger evaluation of a pilot feasibility trial of a PMP developed collaboratively by The Body Image and Eating Disorder Treatment and Recovery Service (BETRS) at St Vincent’s and Austin Hospitals, and Eating Disorders Victoria (EDV). The design and procedure of the PMP and results of the larger study have been described in detail [35, 40], with brief details provided here.

### Ethics

Ethics approval for the research evaluation of the above program was granted by the Austin Health Human Research Ethics Committee (HREC/16/Austin/415), the St Vincent’s Hospital Human Research Ethics Committee (HREC 204/16), The Melbourne Clinic Research Ethics Committee (TMC 301), and the Australian Catholic University Human Research Ethics Committee (2017-90R20777).

Upon registration and recruitment, all mentees and mentors (henceforth collectively referred to as participants) provided written informed consent to participate in the research.

### Peer Mentor program (PMP)

#### Participants

Inclusion criteria for mentees wishing to participate in the PMP were: (1) Adults with a current diagnoses of an eating disorder according to the Diagnostic and Statistical Manual of Mental Disorders (DSM-5); (2) transitioning out of in-patient or intensive day-patient programs from one of three Melbourne facilities and; (3) currently actively engaged in medical and/or psychological treatment for their ED throughout the mentoring program.

Inclusion criteria for prospective mentors wishing to participate in the PMP were: (1) Recovery from a previous eating disorder for at least one year and; (2) successful application for a paid casual position with EDV. Diagnoses for all participants were self-declared.

Exclusion criteria for prospective participants was anyone who was at serious risk of harm to oneself or another, as determined by the clinical team.

#### Mentor training

Successful mentors were employed and provided with three days of mentoring training which outlined the aims, policies and procedures of the program, and provided guidance on managing boundaries and risk. EDV offered ongoing support to mentors in the form of follow-up phone calls, and three group supervision and peer support sessions facilitated by a mental health care professional.

#### Matching process

Mentee/mentor pairs were matched where possible by EDV based on mentees’ preferences (e.g., mentor diagnoses, age, gender, and interests) and logistics (e.g., travel time for meetings) which were ascertained upon registration [19]. Mentors provided a short statement about their values and work style to facilitate effective matching. At least two preferences were met for each participant upon matching. In the event that a matched participant withdrew from the program, their partner was re-matched as soon as a suitable partner became available.

#### Program description

The program comprised of 13 three-hour sessions in community settings which were conducted over a three-to-six-month period (i.e. some pairs met weekly, while others met fortnightly). Activities for sessions were planned collaboratively with individually-tailored recovery goals in mind, and included daily living tasks, practicing social interactions involving food, and making connections in the community. Mentors were encouraged to share their own story of recovery to foster hope and provide empathic support.

Support was provided to mentors and mentees by mental health professionals and program staff from EDV. Group sessions (for mentees and mentors separately) were offered bimonthly, while phone support was offered as required or indicated. Further details of the program, including full inclusion/exclusion criteria, mentor recruitment, training and employment arrangements are accessible in [35, 40].

### Research protocol

#### Recruitment

Participants of the PMP were invited to undertake a semi-structured interview at the cessation of their involvement in the program. Recruitment for this component of the research project concluded once thematic analysis indicated the qualitative principle of data saturation had been reached (in line with the recommendations of Fusch and Ness [41]). Hence, not all of the 30 mentees and 17 mentors from the broader research project were included in the qualitative aspect of the evaluation.

#### Materials

*Online reflections* (presented in supplementary material, Additional file 1) were delivered via an email link, and consisted of a series of open questions regarding the experience of the mentoring program. Both groups (mentees and mentors) received similar questions, although wording was slightly tailored to each group. Questions were designed to explore perceived benefits and challenges of participation, how mentoring differed from traditional treatment, and suggested improvements for the program.

*Semi-Structured Qualitative Interviews.* A semi-structured topic guide (supplementary material, Additional file 2) consisting of open-ended questions was used to generate unbiased and open discussion among participants during the interviews. The topics chosen reflected the aims of the study and were designed to explore themes identified in the empirical literature as relevant to participating in a peer mentoring program, such as unique challenges and benefits [23, 42], as well as themes raised by participants through the online reflection exercises.

#### Procedure

Participants were asked to complete the online reflection at three time-points: After session one (baseline session), at the half-way point of the PMP, and at the completion of mentoring sessions. At the completion of the program, semi-structured interviews were conducted. Interviews took place at EDV or over the phone when necessary. Participants were reminded of confidentiality and de-identification of data at the commencement of each qualitative interview. Interviews averaged 64 min (*range* = 18–74 min) in duration, were audio-recorded, and transcribed verbatim to prepare for analysis.

#### Data analysis

The data from the online reflections and transcribed interviews were imported into NVivo [43]. Data were analysed using thematic analysis. Thematic analysis engages inductive reasoning and involves searching across all the data for repeated patterns of meaning, called themes [44]. Data were first coded into common categories of reported experiences, then these codes were collapsed into larger themes with no a priori theoretical assumptions applied. In an iterative process, each theme was then evaluated regarding the appropriateness of the coded statements they contained. Adjustments were made at the appropriate level (e.g. the theme may be adjusted, or the data re-coded). Finally, themes were interpreted in light of the broader meaning and significance they offer to the research question and the reviewed literature [44].

## Results

### Mentees

Fourteen mentees (13 women) aged 19–52 (*M*_age_ = 30, *SD* = 12), participated in the qualitative study. Of these, 50% were employed, 15% were unemployed, 30% were students, and 5% did not indicate employment status. Thirteen mentees reported a primary diagnosis of (AN), and one indicated a primary diagnosis of Other Specified Feeding and Eating Disorder. The duration of illness amongst mentees ranged from two to 30 years (*M* = 12; *SD* = 11; Median = 6).

Eleven of the 14 mentees who participated in the qualitative evaluation completed the PMP and three withdrew before completion. Of the withdrawn participants, one reported their busy schedule was the primary reason for withdrawal, but also stated that the relationship felt “forced”. The second reported they were not well matched with their mentor in terms of personality and found an imbalance of sharing within the relationship. This mentee also reported having high expectations from the outset. The third reported benefiting from and enjoying the program, however withdrew due to increased social anxiety preventing them from leaving the house.

### Mentors

Mentors were 12 adults employed by EDV (age range 26–39, *M* = 29.36, *SD* = 4.30) with a past history of an ED (11 females, 1 male). One mentor interviewed withdrew from the PMP program before completion due to personal reasons and the six-week gap between training and the assigning of a mentee,^1^ but was included in the current evaluation. Previous primary diagnoses were AN (*n* = 10) and Bulimia Nervosa (*n* = 2). Mean duration of illness was 6.65 years (*SD* = 4.01), and mean duration of recovery was 3.95 years (*SD* = 2.21).

Throughout the reporting of results, all participant names have been substituted with pseudonyms to protect privacy and confidentiality.

### Mentee results

**Fig. 1:**
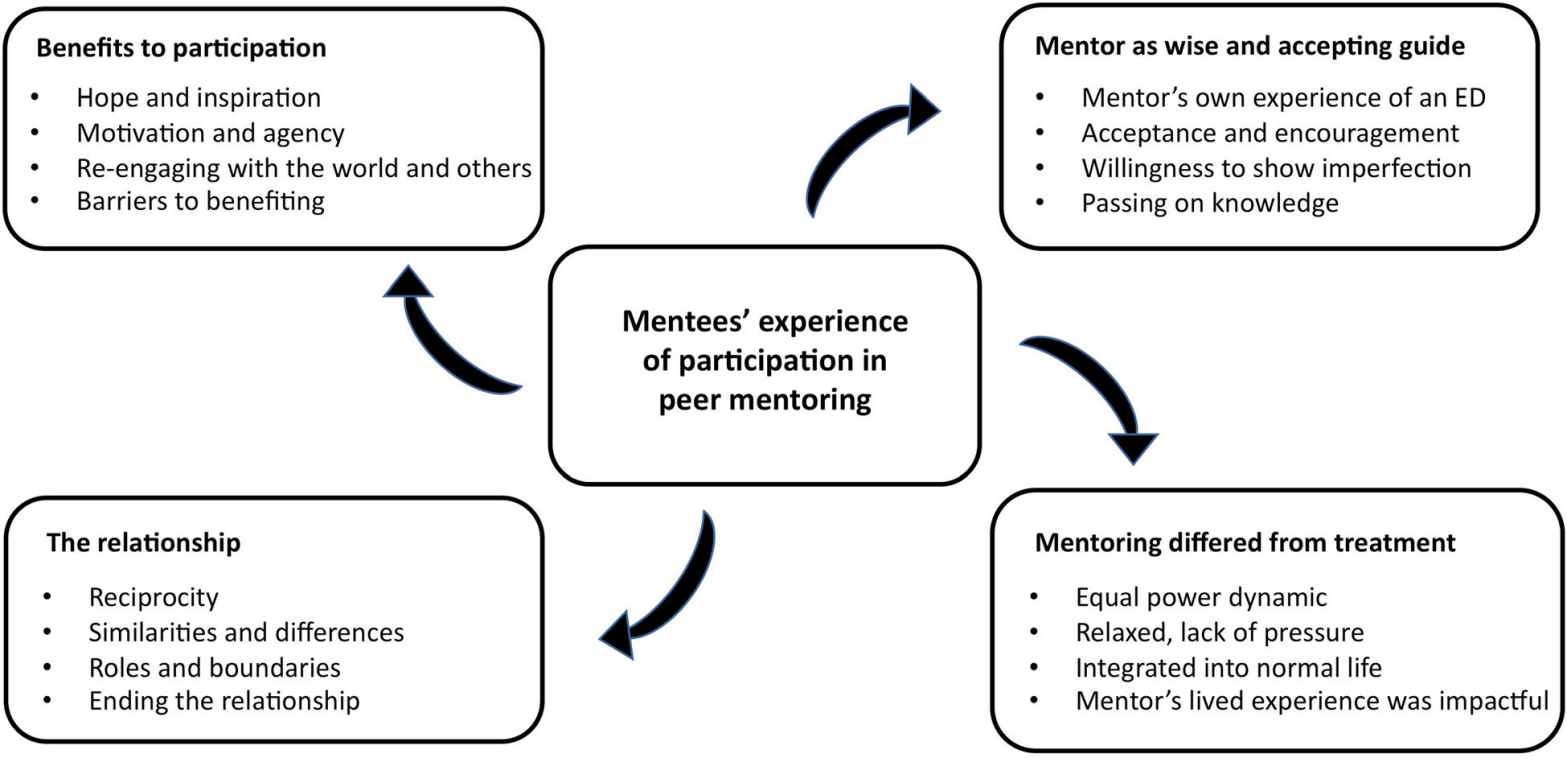
Themes and subthemes of mentee experiences

Thematic analysis revealed four themes relating to the mentees’ experiences of participating in the PMP. These themes and associated subthemes are displayed in Fig. 1.

#### Theme one: benefits to participation

Theme one describes the benefits that mentees received from participation in the program and is broken down into four subthemes. First, *hope and inspiration* describes how mentoring inspired a greater belief that recovery is possible. Witnessing their mentors’ successful recovery increased hope that they too could be freed from the cycle of chronic illness and relapse. One 19-year-old participant (Talia) commented that since meeting her mentor, she was no longer “scared of [her] twenties”, which she had previously assumed would be characterised by illness and isolation. Another participant, Michelle, stated: “I think it was just inspiring that no, actually this is a life that maybe I do want more than I want to be sick.”

*Motivation and agency* describes an increased readiness for action. Margie described: “My brain is starting to recognise that healthy, happy, is actually worth something.” Mentees reported that their involvement with mentoring motivated them to commit to recovery-focused actions, included self-care activities, social eating challenges and reaching out to friends. Michelle offered: “It was nice to have someone to…go and do activities with and kind of give things a go and have a laugh along the way.” Nicole reported: “I got in touch with one of my really close friends who doesn’t live too far away and…[asked], you know, ‘If I could… if I need to talk to someone, could I talk to you?’”

The next subtheme is *re-engaging with the world and others.* Mentees typically described their illness as profoundly isolating. Mentoring provided a bridge to support the transition back to a more connected and “normal” life. The mentoring sessions were an opportunity to socialise free of the imperative to pretend they were well, and a chance to increase tolerance for leaving the house and exploring being out and about. Jasmine said: “I just have anxiety around crowded restaurants…and at the start we’d be like, let’s go somewhere else, but now I can just deal with it.”

The final subtheme explored the perceived *barriers to benefiting* from the program. Despite being prompted to reflect on negative aspects of the program, few barriers or challenges were identified by participants. However, not all mentee experiences were positive; three mentees withdrew from the current program before completion. Taken together, the stated reasons (reported above) draw attention to two main factors. The first is the importance of a natural and complementary connection in the mentoring relationship, which is explored further under *Theme three: The relationship*. The second factor was the importance of a certain level of readiness for participation, including a minimum wellness level, ability to make time and space for the program, and adopting realistic expectations. Julia blamed herself for not benefiting from the program due to her self-reported high expectations, commenting: “It was probably more because of me that I didn’t get much out of it...I went in thinking that this person could give me a road map [for recovery]”. Another withdrawn participant (Sarah) reported enjoying the program, but stated an exacerbation of her social anxiety made it too difficult to attend. Helen reported struggling to juggle work, medical, and mentoring commitments. Two participants who completed the program reflected that they could imagine participation at an acute stage of the illness would not be helpful, due to that stage being characterised by an unwillingness to explore alternative ways of being.

#### Theme two: Mentor as a wise and accepting guide

Most mentees described their mentors in a spirit of great admiration and enthusiasm. The key aspects of the mentoring role are described through four sub-themes. First, the *mentor’s own experience of an eating disorder* emerged as the foundational feature that allowed the mentor’s other qualities (described in subsequent subthemes) to be effective. Nicole reported: “I believe these silly little rules, and…[my mentor] could really properly understand it all.” This concept was endorsed by all but one (withdrawn) participant, who reported she didn’t see value in her mentor’s past experience of an ED. Second, the mentor’s *willingness to show imperfection* offered permission for the mentee to do the same, thus promoting openness and trust. Michelle stated: “My mentor was extremely upfront and honest and…just really real as well.” Relatedly, the mentor displayed acceptance of wherever the mentee was “at” in their illness, and this *acceptance* facilitated provision of unconditional *encouragement*. Rene commented: “It’s just so nice to talk to someone who wasn’t going to judge you, and just accepted it, and if you had a bad week, that’s ok, you know, keep moving forward, keep going.” Margie appreciated that her mentor “really pointed out things that I was doing well.”

Mentees described their mentor in a role akin to a teacher or guide, *passing on valuable knowledge* about the nature of recovery. They offered practical strategies and provided helpful perspectives on life and relationships more generally. Michelle said: “My mentor gave me some sayings and things to do when those [difficult] times came up, and even book suggestions.” In contrast, Julia, who withdrew from the program, reported she received impractical advice from her mentor, such as “listen to your body”. She commented: “[It was] not very specific advice, or concrete I guess. It was a lot more about feelings and emotions, and that wouldn’t really help me.” She reflected this may have been due to her mentor forgetting the finer details and practical tips that helped her in her own recovery. However, the majority of mentees reported their mentor’s advice and support was valuable. The following quote embodies the descriptions of mentors acting as a guide on their mentee’s recovery journey.

*It was like she’d come down the mountain a little and throw me a rope, and it would pull me up a little bit every time… then the next time she didn’t have to come as far down the mountain…It was almost like we were getting to a point of meeting in the middle. But the middle wasn’t actually the middle of the mountain, it was closer to her top than to my bottom*. –Margie.

Most mentees described their mentor as someone who offered permission to be just as they were, whilst simultaneously helping them move forward. Maintaining this delicate balance facilitated the mentee opening to new knowledge and perspectives about recovery and life in general.

#### Theme three: the relationship

Mentees explained the quality of *the relationship* formed with their mentor as being vital to their engagement in the program. The third theme has four associated subthemes. Successful pairings maintained this connection through *reciprocity* (finding mutual value in spending time together) as illustrated by Jackie: “It’s a two-way street thing…I did find [it] comforting to deal with I guess, not feeling so spotlighted, not feeling like centre stage and…we did share a…lot of personal things.” Having personal *similarities* as well as appreciation for *differences* was also important. Jasmine said: “I was surprised we had a lot in common, we spoke a lot about, like environmental things like feminism and politics and stuff. It was nice”. Michelle commented: “The way [my mentor] thinks about things is almost the polar opposite to me, so that was quite helpful.” Unsuccessful dyads either did not appear to have many similarities, or there was a lack of perceived value in the differences between them. One of the three withdrawn participants, Julia, stated the relationship didn’t flow: “just because we were different people I guess–we didn’t really seem to connect.” A mutual understanding and respect of *roles and boundaries* was identified as crucial to a successful pairing. Andrea commented: “I think [my mentor] had really good boundaries in terms of the types of things they talked about.”

The completion of the program brought with it the *ending of the relationship*, which was commonly experienced by mentees as a difficult and sad separation, heralding the loss of an important support person.^2^ This was the most frequently endorsed challenge associated with participation in the program. Jackie reported: “I was quite sad at the end of it…it felt like we’d formed a friendship and…I found it quite sad that we wouldn’t have the opportunity to keep going with that.” Some mentees described the end of the program as representing another transition in their recovery, and was associated with a temporary set-back and re-emergence of symptoms. Fiona commented: “It felt like just another transition to deal with, and probably that would be my main critique of the program.” Others reported that they explored with their mentor ways to build their support network elsewhere after the program, to ensure the loss of support did not result in a drift into old patterns. A few mentees reported that they did not feel prepared for the separation because the policy prohibiting contact after program completion had not been sufficiently explained by program coordinators.

#### Theme four: mentoring differed from treatment

All participants endorsed mentoring as a qualitatively different experience from clinical treatment. Many of the four subthemes described here overlap with previously discussed themes. It was nonetheless considered useful to create a separate theme specifically to delineate the ways in which mentees characterised mentoring and clinical treatment differently, because there is little literature published on this topic.

Most mentees described mentoring as allowing a more collaborative and *equal power dynamic*. Talia reported: “I felt like I didn’t have any power [in treatment] because it was that weird dynamic between a doctor so it was nice to do something that’s supposed to be therapeutic, but it could be more of a friend, equal.” Mentoring was described as being more *relaxed* with a *lack of pressure*. Rene commented: “There was none of that pressure [compared to clinical treatment], and I felt really good about that – because it actually made me want to try harder the next time.” Mentoring was characterised as being more *integrated into normal life*. Jordan stated: “You can do a lot of things [in mentoring]. You can’t just go…for example grocery shopping with a psychologist.” Finally it was described as having more *impact due to the mentor’s shared experience* of an eating disorder. Jasmine described that advice “has a different kind of impact coming from some one who has been through it.”

Of note, whilst discussions around this theme tended to be framed by mentees in terms of a *comparison* between the two modalities, it was acknowledged by several mentees that the two complement each other, and both had a unique and necessary role to play in supporting them through recovery. Michelle said: “I know a peer mentor is not considered treatment in the normal sense, but it complements treatment and I think it’s one of the best things I’ve done.”

### Mentor results

**Fig. 2:**
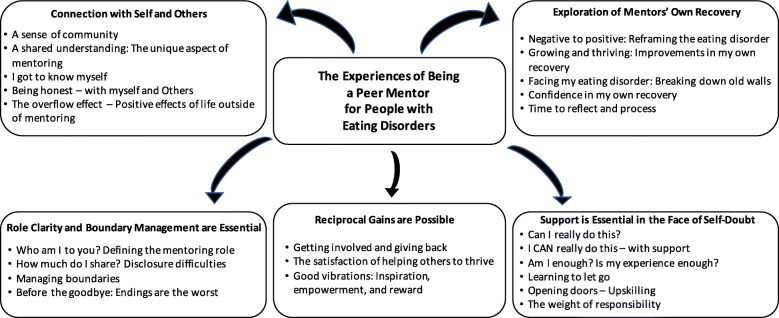
Themes and subthemes of mentor experiences

Five key themes emerged from the online mentor evaluation forms and the qualitative interviews. Each theme is presented with corresponding subthemes in Fig. 2.

#### Theme one: connection with self and others

The first theme captures the sense of connection with self and others within the ED community that was ubiquitous across all 12 mentors. Mentors emphasised a *sense of community* as one of the cornerstones of the positive mentoring experience, with many expressing the power of “just getting 15 or so people who’d been through EDs in the same room” (Jamie, when discussing the group supervision sessions). The power was attributed to the fact that many mentors had not knowingly met another recovered individual before and were now doing so with a sense of purpose and positivity – “It was great to meet a lot of other people who had recovered” (Tanya). Most mentors considered the unique aspect of mentoring to be *a shared understanding*; or “not feeling like you have to explain [your experience] or explain yourself” (Kate). Through this shorthand, mentors believed they were able to” connect on a really authentic level” (Kate) and build rapport quickly. Five mentors noted that the PMP experience allowed them to better connect with themselves, stating that “there is an integration of your own self that happens [in the process of mentoring]” (Jamie) through self-reflection and meeting others who are similar in certain ways – “Before you even go through the door you know you’ve got something in common, there’s a shared connection there” (Sandy). This integration of self was also intertwined with (and reliant on) a sense that *being honest with myself and others* was essential in the mentoring process; Kim pointed out that “the more you admit that [you’re not perfect], the more everyone gets from it”.

#### Theme two: exploration of mentors’ own recovery

The second theme details the recurring sense that mentors were able to explore their own recovery through the PMP. Most mentors considered that the PMP had allowed them to *reframe the eating disorder experience from negative to positive*, determining their experience to be “valuable now…actually worth something, other than just suffering and confusion” (Jamie). Mentors also noted that the PMP unexpectedly resulted in *improvements in their own recovery* though a process of reciprocity, with Kim commenting that “the more I give, the healthier I am, like the less I think about myself is the healthiest place for me”,and Steph noting “[the PMP reinforces] everything you learn in recovery”. The mentoring process meant that there was a need for mentors to *face their eating disorder*, which for some led to the realisation that “[a] veneer…had grown between me and those experiences…I just [had] this wall…and I couldn’t have that when I was mentoring”(Rory). Through this confrontation of their ED, some mentors built *confidence in their own recovery*, experiencing “a big relief…thinking that I can still…reframe this…like I’m not just going to live my whole life fearing…it” (Tanya). One of the greatest benefits of the program was reported to be a chance “to reflect on how incredibly far I have come” (Jamie) through an *increase in perspective on the ED journey*, which also led to revelations that **“**there’s never really 100% recovery - it’s always 95 and there’s the 5% that you…keep trying to improve” (Taylor).

#### Theme three: support is essential in the face of self-doubt

The third theme captures the common reports of self-doubt in the early stages of the PMP, followed by an increase in confidence as the program went on. Before training and through early mentoring sessions, most mentors wondered *can I really do this?* with skills-based concerns that “I wouldn’t have enough to say and do the right things” (Kim)” and more personal worries including “if someone [triggers me]-how do I deal with that” (Sandy). Sandy also reported that although communication received from EDV at this stage was sufficient to placate any concerns about their competency, there was “anxiety as to whether I would be paired up with anybody”, which was also echoed by another mentor who withdrew at this stage who reflected that had they been matched faster, they may have remained in the program. Mentors also felt *the weight of responsibility* at times*,* with Jordan commenting that a decline in their mentee’s functioning left her “questioning whether or not [she] had done enough”. Fortunately, early anxiety over inexperience, skills, and the emotional weight of mentoring were mitigated in the first instance via pre-program training, allowing mentors to “mentally prepare for who we would be working with” (Jules). This continued later through group supervision with other mentors, which was found to be “really valuable, just to…get…ideas-and…understand certain situations” (Rowan). Training and supervision were critical factors in mentors developing a sense of *I can do this*, and also resulted in the *opening of new doors*, both personally and professionally as mentors gained “experience and confidence in the [mentoring] role” (Jules) with “opportunities to learn… and build skills” (Kate).

#### Theme four: reciprocal gains are possible

The fourth theme details the mentors’ hopes before commencing their role, and the often-surprising gains which occurred during the PMP. When discussing their reasons for applying for the PMP, almost all (10) mentors cited a desire to *get involved and give back*, seeing the program as “a great way to share my experience and help other people” (Rowan). Whilst some mentors had spent many years avoiding anything to do with EDs until “something clicked…and I just felt ready to bring [my experience] back into my life” (Tanya), others felt they had “wanted for quite a long time…for there to be…some platform in which I could use my experiences to…help someone else” (Steph). When considering the personal impact of the program, mentors commented on the intrinsic sense of *satisfaction of helping others to thrive*, whereby they walked away from their sessions “[feeling] like [I am] making a difference…having a positive influence on somebody else” (Tanya) as well as the opportunity “to just be free and to be able to just discuss whatever and not be afraid of being judged” (Taylor)*.* Mentors also expressed their surprise at how much they gained from their time with their mentee, considering many reported coming into the program for mostly altruistic reasons. Reports of *inspiration, empowerment, and reward* were common, with frequent accounts of “feeling really inspired by my mentee” (Kate).

#### Theme five: role clarity and boundary management are essential

The fifth and final theme captures some of the most commonly identified challenges of the program for mentors. *Defining the mentor role* was considered by many to be a significant challenge due to the task of “finding a balance between…necessary detachment…and [striving for] the equally-necessary intimacy created by and needed for a solid connection” (Rory)*.* For most mentors, part of the role-delineation process involved *defining and managing boundaries* and building of “open conversation, respect, understanding, and appropriate support” (Rory). Aspects of training found to be most helpful by mentors included “learning…how to say “no”” (Jamie) and EDV’s emphasis that mentoring was ““only in business hours”…"only…through [certain methods of contact]”” (Rory). The final–and ultimate–test of boundary management for most mentors was almost unanimously considered to occur *before the goodbye,* during the final mentoring session. Reasons for this difficulty ranged from trying to balance sending a firm message of ““this is 13 sessions, [it] is what it is”, and making sure that didn’t come across as a rejection” (Steph), and the possibility that “after ending the mentoring relationship [a…mentee’s] condition may decline” (Jules). Findings indicate that mentors dealt with these difficulties using EDV’s support to “[guide] me through that process of how to end that relationship…talking about…ways to potentially discuss it with my [mentee]” (Kate), and through “[reminding] myself that my participant has the strengths and skills to continue with…recovery without the mentoring program” (Jules).

## Discussion

This qualitative study aimed to investigate the benefits and challenges experienced by mentees and mentors in a peer-mentoring program for individuals in early recovery from an eating disorder. The secondary aim was to explore (from the mentees’ perspective) the experiential difference between receiving support through peer mentoring as opposed to clinical treatment modalities.

Hope, which is closely linked to motivation [45], has been identified as an important aspect of recovery in mental illness generally [46, 47] and eating disorders specifically [11, 16]. The cultivation of hope is perhaps the strongest common finding in the limited existing research examining peer mentoring programs for individuals with ED [36–39]. In parallel to the current study, Ramjan et al. [39] found that mentor’s positivity, strength, and resilience was experienced by mentees as tangible evidence of the possibility of recovery. Also in consensus with the current study, Ramjan et al. [39] reported that mentoring supported mentees in “reconnecting with the world”, as mentors were uniquely situated to provide a social context that was honest and open, while simultaneously challenging. Relatedly, Lippi [36] noted that mentoring offered mentees the opportunity to re-learn social skills in a safe context. The data in this study suggests these social benefits were afforded to those mentees who enjoyed a connected and harmonious mentoring relationship. The matching process attempted to ensure this outcome for all mentees, by offering them opportunity to select matching criteria of most importance to them. However, the restricted size and diversity of the mentor pool as well as (often unknown) pre-existing interpersonal patterns and preferences limited the assurance of achieving successful relationship pairings in all cases.

Mentoring was consistently perceived by mentees as a more relaxed form of recovery support than clinical treatment. The elements of spontaneity and fun reported by mentees in the current evaluation was also found in Lippi’s [36] study. These elements may be particularly important in eating disorder treatment, due to the high level of monitoring and directiveness necessary in clinical treatment, which can be perceived as highly stressful and controlling [48].

Echoing results found in the current study, mentors in Ramjan et al.’s study [39] reported that delivering mentoring facilitated the transformation of a negative experience into a positive one, through supporting and sharing their story with others. Indeed, altruism and the sharing of a traumatic experience have been described as powerful stimulators of self-healing [39, 49]. The current results also align with reports from mental health peer mentoring more generally, which demonstrate multiple benefits to undertaking peer support work, including an increase in self-esteem and confidence [19, 28], an opportunity to reframe negative experiences [11, 29], individual and professional growth [30, 36]; as well as the opportunity to get involved and give back [50, 51].

Mentors in the current study experienced an increase in eating disorder symptoms across the program (reported in the quantitative data [40]), whereas the mentors in Ramjan et al.’s study [38] did not. The insights gathered from the qualitative data did not contribute to a direct explanation of this occurrence. Nonetheless, some negative experiences discussed by mentors may help elucidate risks to delivering mentoring. Negotiating boundaries was reported as a significant challenge in this cohort and is widely recognised as particularly difficult in mentoring. The expectation of caring for their mentee in addition to engaging in self-disclosure complicates the role [19, 39], encumbering mentors with both professional responsibility and personal vulnerability. The mentors in Ramjan et al.’s study [39] delivered less hours of face-to-face support than mentors in the current study, as a significant proportion of the program was delivered via digital communication. The more direct form of support delivered by mentors in this study may have provided opportunity for a comparatively higher levels of disclosure, vulnerability and responsibility in their role. Research suggests that as relational vulnerability increases with personal disclosure, so too does the risk of job stress, burnout, and relapse [18, 19, 31]. Mentees in this study indicated that their mentors managed this balance successfully from their (mentee) perspective at least. In the words of Solomon [47], it could be said that these mentors were “expert at not being an expert”. However, mentors indicated this significant balancing act was not always achieved with ease, and they concurred that the training and support provided by EDV was critical to managing this challenge.

The most commonly reported challenge of participation reported by both mentees and mentors was the sadness and loss due to separating from their mentor at program completion. Previous mentoring literature noted that there can be difficulties with ending the mentoring relationship due to the bond that often develops [51]. The difficulty associated with gaining then losing a significant source of support (for mentees) and a meaningful relationship (for mentors) should be carefully considered as a potential hindrance to balance against potential gains.

Much of the research on peer-mentoring emphasises the importance of supervision and training of mentors [19, 52, 53] and the (mainly) positive experiences reported by participants in this study should be considered in the context of a program that offered mentors extensive support when the strain of the role was felt. It should be noted that the degree of training and supervision offered in this program may be difficult for organisations to replicate outside of a funded research project, and this may be a barrier to safe and effective delivery of peer mentoring programs. The withdrawal of one mentor was in part due to the length of time between initial training and subsequent matching with a mentee, which could indicate that confidence gained in training and supervision may diminish if acquired skills are not put to use in a timely manner. Other mentors experienced anxiety surrounding the anticipation of being matched, indicating that the period between training and matching may be worthy of additional targeted support in future peer mentoring programs.

### Limitations

One of the primary aims of the research was to understand the challenges associated with participation, however, few challenges were reported by participants. Self-depreciation and a desire to please others are common personality traits for those with eating disorders [54, 55], which may have led to *social desirability response bias* [56], thus masking more accurate critical feedback of the program despite efforts by the researchers to encourage directness. This may have impacted the ability of the qualitative data to explain the mentor’s increased eating disorder symptoms or accurate reasons for mentees’ withdrawal.

The current study used a heterogenous sample, consisting of individuals with varying ED diagnoses and genders. Experiences of illness and recovery are not consistent among these different groups ([13, 57], respectively). Modifying inclusion criteria to a homogenous group (e.g. individuals with BN, or an all-male or female mentor pool), would allow investigation of the specific mentoring needs of that particular sub-group, and the role of gender. Of note, no difficulties related to differences in diagnosis or gender within the mentoring relationship were reported by participants in this study.

### Future directions and implications

Emerging patterns across the current and previous studies suggests mentoring may play an important role in assisting an individual in early recovery to cultivate hope and to ‘meet the world’ again in a safe, enjoyable, and accepting social context. Importantly, the relaxed, fun and social nature of mentoring may complement the more structured and formal nature of clinical treatment models. These findings offer treating clinicians pause to reflect on the importance of striking a balance between communicating acceptance towards their clients’ current state, and facilitating forward movement in treatment.

The current study suggests benefits for mentees and mentors are most likely to be realised in the context of adequate and ongoing training, supervision, and support. As navigating separation is an under-researched facet of peer-mentoring research, literature relating to termination of the therapeutic relationship [58] may be an appropriate source of guidance to draw upon to mitigate negative impacts of this loss for participants in future programs. This content should be introduced from the mid-point of the program to allow ample time for the dyad to prepare for separation, and to cultivate (mentee) support networks external to the program. Mentors may also benefit from targeted support during the time between training and matching, to lessen the potential of anticipatory anxiety leading up to mentoring.

Given the encouraging results of this study and other similar programs [e.g. 39], future efforts should include a controlled trial using a *treatment as usual* comparison group and include long-term follow up, so that outcomes such as effect size can be examined over time.

## Conclusions

This study contributes to the small and growing body of evidence suggesting value in pursuing peer mentoring as one part of a comprehensive approach to eating disorder treatment. Results indicate that peer mentoring has the potential to provide unique and beneficial support to complement clinical treatment, warranting further research efforts and program development.

## Supplementary information

**Additional file 1: Appendix A.** Online Reflection Questions.

**Additional file 2: Appendix B.** Interview Schedule.

## Data Availability

The datasets generated and analysed during the current study are not publicly available, but may be available from the corresponding author on reasonable request.

## Abbreviations

AN: Anorexia nervosa
BED: Binge Eating Disorder
BETRS: Body Image and Eating Disorders Treatment and Recovery Service
DASS: Depression Anxiety Stress Scale
DSM-5: Diagnostic and Statistical Manual of Mental Disorders, 5th Edition
ED: Eating disorder
EDNOS: Eating Disorder Not Otherwise Specified
EDV: Eating Disorders Victoria
OSFED: Other specified feeding and eating disorder
PMP: Peer Mentor Program
TMC: The Melbourne Clinic

## Footnotes

1 Due to the small sample being known to each other, further details regarding reasons for withdrawal may compromise participants’ anonymity and have therefore been withheld.

2 PMP policy did not permit contact between the mentee and mentor after program completion.
