# Supplementary material for: Peer mentoring for individuals with an eating disorder: a qualitative evaluation of a pilot program
Source: J Eat Disord. 2020 Jul 1;8:29. doi: 10.1186/s40337-020-00301-8 (PMC7329554; doi:10.1186/s40337-020-00301-8)
Supplement: Supplementary file 1 — Additional file 1: Appendix A. Online Reflection Questions. [file 40337_2020_301_MOESM1_ESM.docx]

Appendix A

Online Reflection Questions


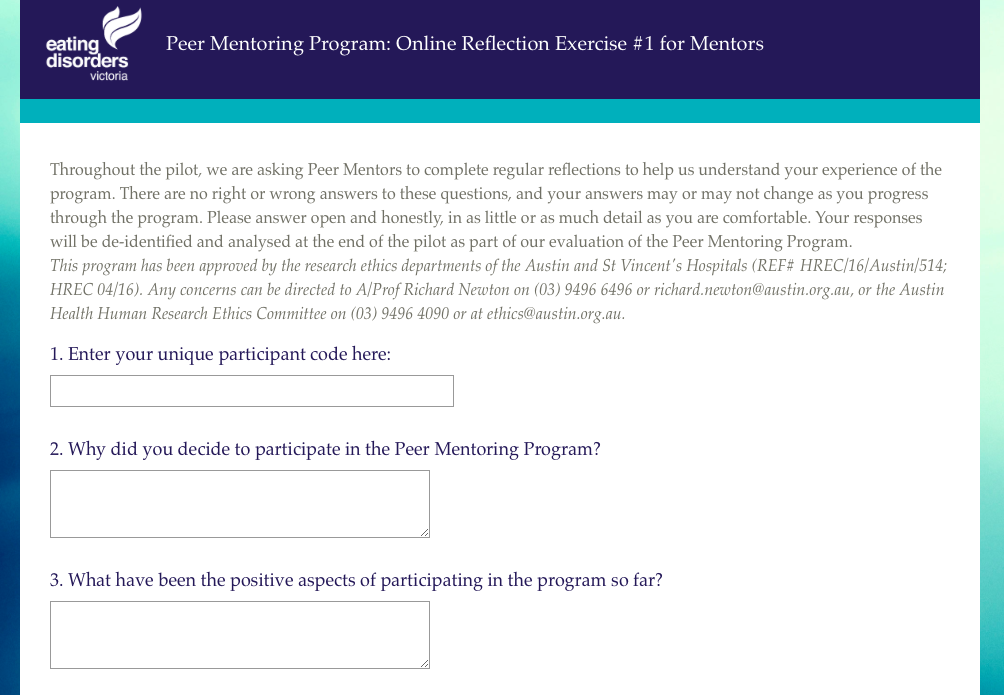


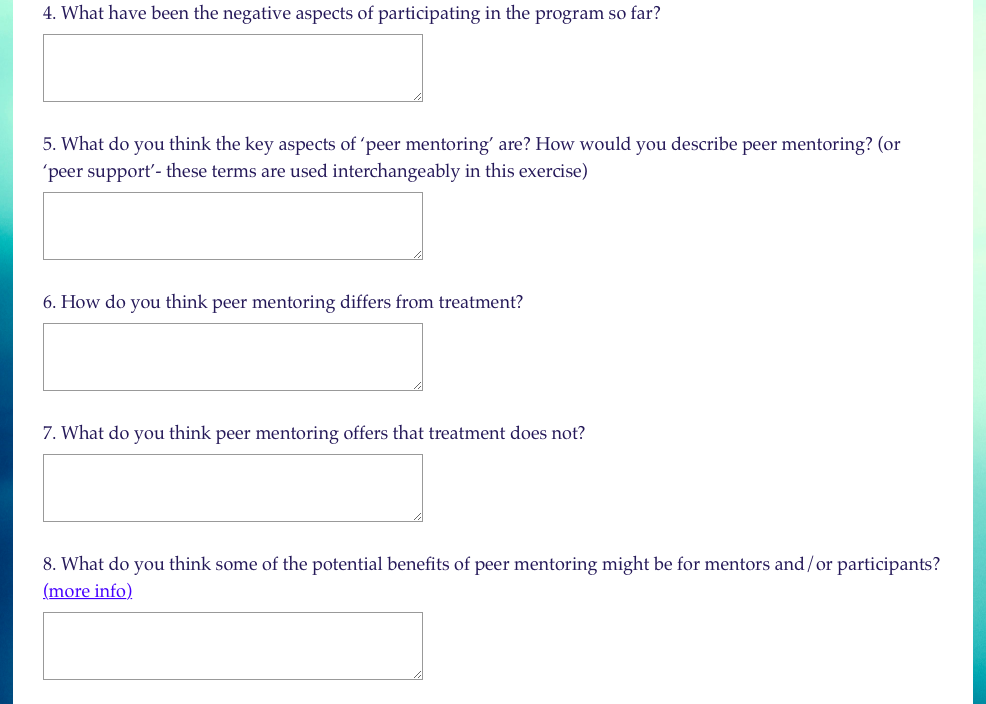


*Note.* Example is taken from *Mentor* form. *Mentee* form is identical in content but varies in wording to address mentees.
