# Supplementary material for: Peer mentoring for individuals with an eating disorder: a qualitative evaluation of a pilot program
Source: J Eat Disord. 2020 Jul 1;8:29. doi: 10.1186/s40337-020-00301-8 (PMC7329554; doi:10.1186/s40337-020-00301-8)
Supplement: Supplementary file 2 — Additional file 2: Appendix B. Interview Schedule. [file 40337_2020_301_MOESM2_ESM.docx]

Appendix B

Interview Schedule

Participant’s Experience - General

- What was your experience in participating in the mentoring program?
- General
- Question/s regarding the mentoring program
- Decision to participate

Positive

“What where the positive aspects of participating in the mentoring program?”

1. Potential perceived benefits to mentees/mentors
   1. Recovery
   2. Re-admission
   3. Social support
   4. Social functioning
   5. Empathy and acceptance
   6. Reducing stigma
   7. Increasing hope

Negative

“What where the negative aspects of participating in the mentoring program”?

1. Potential perceived challenges to mentees/mentors
   1. Boundaries
   2. Power
   3. Stress
   4. Accountability
   5. Maintaining the role
2. Potential barriers to participation
   1. Logistics (e.g., travel distance. financial difficulties)
   2. Illness
   3. Embarrassment/shame

Participants’ Perception of Peer Mentoring

Why did you decide to participate in the mentoring program”?

1. Definition of peer support
2. How it differs from treatment
3. What it adds to treatment

Other

1. Response of treatment team
2. Suggestion for future improvements
3. Any other relevant factors perceived by the participant as relevant/important to attendance
